# Poverty Simulations: Are the Learning Outcomes Consistently and Uniformly Positive?

**DOI:** 10.1002/jdd.70090

**Published:** 2025-10-26

**Authors:** Michelle R. McQuistan, Lance Brendan Young, Chandler Pendleton

**Affiliations:** ^1^ Department of Preventive and Community Dentistry The University of Iowa College of Dentistry and Dental Clinics Iowa City Iowa USA; ^2^ Iowa Institute for Oral Health Research University of Iowa College of Dentistry and Dental Clinics Iowa City Iowa USA; ^3^ Florida State University College of Medicine Tallahassee Florida USA

**Keywords:** access disparities, behavioral sciences, community‐based education, doctor/patient relationship, educational methodology, oral health disparities, patient‐centered care, socioeconomics

## Abstract

**Objectives:**

Research indicates significant improvement in average attitudes toward those in poverty following poverty simulations, but little research addresses whether students benefit uniformly. This study measured variability in poverty attitude change following poverty simulations and tested whether poverty attitudes are associated with simulation evaluations.

**Methods:**

Third‐year dental students were surveyed at baseline (T_1_), post‐simulation (T_2_), and 2‐month follow‐up (T_3_). Poverty attitudes reflect Likert‐scale agreement with 20 statements addressing personal responsibility and public policies. Simulation evaluations were measured at T_2_ and T_3_ by agreement with eight statements addressing the simulation's value and impact on patient care. Data analysis relied on descriptive statistics, paired‐samples *t*‐tests, and Pearson correlations.

**Results:**

Students’ (*N* = 169) average poverty attitudes were 9.4 points higher at T_2_ than T_1_ (p < 0.001) and 7.9 points higher at T_3_ than T_1_ (p < 0.001). However, 21.7% reported lower attitude scores at T_2_, and 34.6% reported lower attitude scores at T_3_, with a 4‐point median decline from T_1_ in each group. Baseline poverty attitudes did not predict simulation evaluations, but T_2_ poverty attitudes correlated with T_2_ (*r* = 0.33) and T_3_ (*r* = 0.45) evaluations. Also, T_3_ poverty attitudes were correlated with T_2_ simulation evaluations (*r* = 0.35) and T_3_ (*r* = 0.44) evaluations (all *p* < 0.001).

**Conclusions:**

Poverty simulations enhance poverty attitudes on average, but potentially worsen attitudes among a substantial percentage of students. Those with negative post‐simulation attitudes toward poverty also are likely to value the simulation less and may require different teaching strategies.

## 1 Introduction

In for‐profit health care systems, financial resources are a primary determinant of oral health status [1] and oral health disparities [2]. Further, evidence suggests a linear relationship between socioeconomic status and oral health related quality of life [3]. At the population level, those with the fewest resources have the worst oral health and therefore are in need of the most oral health care.

In the USA, the poorest people may have public insurance options like Medicaid and the Children's Health Insurance Program (CHIP), but an estimated 67% of dentists in the United States do not accept patients covered by these programs [4]. Dentists perceive various systems‐level barriers to their Medicaid participation, including slow reimbursement, low reimbursement rates, and regulatory compliance challenges [5, 6]. They also acknowledge concerns with low‐income patients using Medicaid, including perceptions of more canceled appointments, more complicated treatment needs, and lower treatment compliance [5, 6]. These concerns may have some validity, given Medicaid expansion has not entirely closed the equity gap [7, 8].

Dentists acknowledge an ethical obligation to treat low‐income patients [5], but research shows poorer patients experience significantly less clinician empathy than patients with greater financial resources [9]. Dental school policies like lower tuition and community‐based training requirements are associated with increased Medicaid participation among alumni [10]. Instruction in patient communication and behavioral health have also expanded in dental school curricula in recent decades, with specific efforts to reduce such differential treatment [11]. Further, the Commission on Dental Accreditation (CODA) requires predoctoral programs to meet standard 2‐17, which addresses, “the importance of meeting the health care needs of dentally underserved populations”[12]. Yet, despite these policy and instructional changes, evidence suggests dental students’ willingness to treat low‐income patients remains static [13] or even declines [14] during their time in dental school. A survey at one school found only 39% of graduating dental students plan to participate in Medicaid [15].

Poverty simulations are a curricular strategy designed to increase students’ understanding of poverty and to increase their willingness to treat patients who are struggling financially. These simulations have been a feature of the curriculum at multiple dental schools for years [16, 17, 18, 19, 20, 21, 22]. Survey data over 20 years document improvements in understanding of poverty and attitudes toward poverty following health sciences students’ participation in poverty simulations [17, 23]. These studies, however, overwhelmingly rely on averages of self‐reported attitude scores, without investigating whether the benefits accrue uniformly to participating students. Some research suggests attitude changes may not persist [24], or they may actually change for the worse [25]. It has further been argued that the outcomes research is often methodologically flawed and that poverty simulations themselves may perpetuate inequality [26]. Studies indicate that poverty simulations have a differential influence based on the participant's sex [27], the participant's discipline of study [28], and the participant's status as a student versus professional [29]. Therefore, it is quite possible that although the average attitude improves following a simulation, some participants may leave the experience with a similar or worse attitude than they had before. Research to date has largely ignored these participants.

The objectives of this study were to measure the variability in poverty attitude change following poverty simulations and to determine whether any attitude change is associated with participants’ evaluations of the simulation. Specifically, this study sought to answer three research questions. First (RQ_1_), what percentage of students report more negative attitudes following a poverty simulation? Second (RQ_2_), what percentage of students find poverty simulations valuable? Third (RQ_3_), what percentage of students perceive poverty simulations positively influence their understanding and care of patients?

Further, this study also tested three hypotheses. The first hypothesis (H_1_) proposed post‐simulation improvements in poverty attitudes change significantly two months later. The second hypothesis (H_2_) proposed evaluations of the simulation change significantly 2 months later. The final hypothesis (H_3_) proposed poverty attitudes before and after the simulation are positively correlated with evaluations of the poverty simulation.

## 2 Methods

The Human Subjects Office of University of lowa reviewed, approved, and monitored this study (IRB #200806737). In our predoctoral dental program, the poverty simulation experience is designed to achieve the following learning objectives:

1. To empathize more effectively with patients facing socioeconomic barriers to care.
2. To identify community resources available to patients in poverty and the structural barriers which undermine their effectiveness.
3. To understand the influence of poverty on patient attitudes, behaviors, and perceptions of time and money.
4. To reflect on students’ own levels of cognitive and emotional empathy immediately following the poverty simulation.

The Community Action Poverty Simulation (CAPS) was purchased as a kit from the Missouri Community Action Network [30]. Dental students who participated in the poverty simulation were each assigned the profile of an individual, most of whom belonged to a family struggling financially. Participants were expected to work with family members to meet their social obligations (e.g. school attendance) and financial obligations (e.g. childcare and rent payments) across four 15‐minute “weeks.” Volunteers from the college and community staffed 15 community resources (e.g. employment agency, pawn shop. grocery store, bank, etc.) which could help or hinder the participants’ efforts to remain financially solvent at the end of the month. Debriefings with the participants followed the simulation. The overall learning objective was to better understand the constellation of systemic challenges that can thwart poor families’ efforts to advance economically.

The planning committee endeavored to adhere to the implementation suggestions detailed by the Missouri Community Action Network, although minor modifications were introduced. First, we included the recommended debriefing in which volunteer staffers share their observations about the participants’ behaviors and their personal experiences with actual poverty or with those experiencing poverty. However, as suggested by others [31], we added a half‐hour small‐group discussion period facilitated by faculty or staff members. In small groups students were better able to process their experience in response to prompts and questions designed to elicit insights and relate it to their engagement with dental patients. Second, it is recommended to seek out and compensate volunteer staffers who have experienced poverty as, “an opportunity for low‐income persons to educate the simulation participants” [30]. We did not have the budget to compensate volunteers and we did not explicitly inquire about the financial status of our volunteers. We had found over the years that some volunteers and students who have experienced poverty find CAPS upsetting or triggering. As suggested by other poverty simulation administrators [32], we added language to the volunteer orientation and the Director's Script saying that participants were welcome to share personal experiences, but that it is not expected or required. Some volunteers shared personal experiences of poverty and most shared experiences working with people in financial distress. Every participant from our college had experience with patients facing financial barriers to care because a high proportion of the patient pool qualifies for Medicaid and because our third‐year students had been treating these patients in our preventive clinic for a year. Third, we also included and introduced an on‐site mental health counselor for those experiencing overwhelming negative emotions. Finally, the kit came with a single “Luck of the Draw” card simulating unexpected broken tooth expense, but we added more of these cards to highlight the economic impact of poor oral health on this community.

### 2.1 Participants

The participants were third year predoctoral dental students enrolled in a behavioral sciences course focusing on strategies to address patients’ barriers to care. The course was required, and participation in the simulation was a requirement to both complete the course and to graduate from the program. Students enrolled in the fall semesters of 2022 and 2023 participated in the simulation and the study.

### 2.2 Measures

Attitudes were measured using 20 statements about poverty, addressing personal responsibility and public policies. For example, “People with low income do not have to work as hard because of all the services available to them.” Our instrument is an adaptation of the 20‐item questionnaire which comes with the CAPS kit. It was not a psychometrically validated measure, but it included seven statements from the validated Attitudes Toward Poverty Short Form [33] and six from the validated Undergraduate Perceptions of Poverty Tracking Survey [34]. Response options ranged from *strongly agree* (1) to *strongly disagree* (5), and six items were reverse‐scored. When responses were summed, total attitude scores could range from 20 to 100, with higher total scores indicating a more nuanced understanding of the structural barriers to escaping poverty.

Evaluations of the CAPS experience were measured using eight statements with four response options ranging from *strongly disagree* (1) to *strongly agree* (4). Therefore, a neutral response option (e.g. “neither agree nor disagree”) was unavailable and participants had to lean toward agreement or disagreement. Two statements addressed the value of CAPS participation. For example, “The poverty simulation should be conducted with future classes.” The remaining six statements addressed perceived impact on patient care, including both understanding patients’ financial challenges and intention to treat low‐income patients or those on Medicaid after CAPS participation. When summed, possible total evaluation scores could range from 8 to 32. Although this was not a psychometrically validated measure, we had asked identical questions following CAPS administration in many previous years and believe this measure has face validity.

### 2.3 Data Collection

The CAPS took place on November 15, 2022 (*n* = 84), and November 14, 2023 (*n* = 85). Students completed surveys at three points: baseline (T_1_) within the week prior to CAPS; post‐survey (T_2_) within 3 days after CAPS; and follow‐up (T_3_) approximately 2 months after CAPS. Attitudes about poverty were measured at all three points, but evaluations were measured only at T_2_ and T_3_. In 2022, students were allowed the option of paper surveys or online surveys at T_1_, but all subsequent data collection for 2022 and 2023 simulations was done online using hyperlinks emailed to each student. Qualtrics, the online survey software used, includes a feature which records which students accessed the survey without connecting specific responses to student names. Students were required to access the survey to receive assignment credit for each survey, but were assured their response were anonymous. The advantages of this approach were enhanced candor and survey validity. A disadvantage, however, was that students could gain assignment credit by accessing the survey without completing any responses.

### 2.4 Data Analysis

A measure was discarded if the participant responded to 75% or fewer of the measure's items. Otherwise, case mean substitution was used to replace missing values [35, 36]. Descriptive statistics (frequencies, means, and medians) were used to answer the three research questions. To test whether attitudes and evaluations changed significantly over time, paired‐samples *t*‐tests were used with a 2‐tailed test of significance. Pearson correlations and a 1‐tailed test of significance were used to test for a relationship between attitudes and evaluations at different time points. Significance was established using a value of *p* < 0.05. No adjustments were made for multiple comparisons.

## 3 Results

During the pre‐survey period (T_1_), 169 participants completed the poverty attitudes measure. Of these, 91 (53.8%) were female, 66 (39.1%) were male, and 12 (7.1%) did not specify. Regarding race, 114 (67.5%) were White, non‐Hispanic, 13 (7.7%) were White, Hispanic, 11 (6.5%) were other/multiracial, 10 (5.9%) were Asian/Pacific Islander, 7 (4.1%) were Black, and 14 (8.3%) did not specify a racial category. Also, 115 (68.1%) reported they previously had never or rarely experienced financial insecurity. The number who completed both the attitudes and evaluation measures was 162 in the post‐survey period (T_2_) and 159 in the follow‐up survey period 2 months later (T_3_).

To answer RQ_1_, students who responded to the poverty attitudes measure were categorized based on their attitude change from T_1_ to T_2_ and from T_1_ to T_3_. As shown in Table 1, although the majority reported an improvement in attitude scores from T_1_ to T_2_, 21.6%, a substantial minority, reported poorer attitude scores at T_2_. The median improvement, however, was more than twice the magnitude of the median decline. Two months later, students reporting improved poverty attitudes had dropped 11.8 percentage points compared to baseline, and those reporting poorer attitudes rose 13.0 percentage points to 34.6%. The median improvement for those who improved and the median decline for those with poorer attitudes, however, were identical to the earlier T_1_–T_2_ comparison.

**TABLE 1:** Poverty attitude change from baseline.

	T_1_ to T_2_ *N* = 162			T_1_ to T_3_ *N* = 159		
Attitude Change	Frequency *n* (%)	Mean change	Median change	Frequency *n* (%)	Mean change	Median change
Improved/increased	117 (72.2)	+14.5	+9.0	96 (60.4)	+16.5	+9.0
Did not change	10 (6.2)	0.0	0.0	8 (5.0)	0.0	0.0
Declined/decreased	35 (21.6)	−5.2	−4.0	55 (34.6)	−5.9	−4.0

To answer RQ_2_ and RQ_3_, the frequency and percentage of students who reported *agree* or *strongly agree* with each CAPS evaluation statement at T_2_ and T_3_ were calculated (Table 2). At T_2_, more than three‐quarters of respondents endorsed the two statements addressing the value of the simulation, and both majorities actually increased at T_3_. However, when examining those not in the majority, at the two‐month follow‐up (T_3_) we found 22.5% said CAPS was not beneficial, and 20.6% disagreed that it should be conducted in the future. Regarding impact on patient care, again large majorities ranging from 70.7% to 83.5% agreed at T_2_ with statements that CAPS helped students better understand poverty and their patients struggling with poverty and with statements they would be more likely to treat low‐income and Medicaid patients. Agreement with the enhanced understanding statements remained high or increased at T_3_, but agreement with the statements regarding intention to treat fell. Between T_2_ and T_3_, the proportion disagreeing they were more likely to treat low‐income patients rose 5.1 percentage points to 34.4%. Those disagreeing they were more likely to treat Medicaid patients rose 11.2 percentage points to 39.4%.

**TABLE 2:** Poverty simulation evaluation item responses on post‐ and follow‐up surveys.

	Respondents who agree or strongly agree	
Poverty simulation evaluation item	T_2_ *n* (%) *N* = 164	T_3_ *n* (%) *N* = 160
Value of the simulation		
The poverty simulation was a beneficial activity.	124 (75.6%)	124 (77.5%)
The poverty simulation should be conducted with future classes.	127 (77.4%)	127 (79.4%)
Impact on patient care		
The poverty simulation helped me better understand the financial challenges my patients may face.	129 (78.7%)	122 (76.3%)
The poverty simulation helped me better understand the personal challenges my patients may face.	134 (81.7%)	131 (81.9%)
The poverty simulation helped me better understand the barriers to health care my patients may face.	137 (83.5%)	131 (82.4%)
The poverty simulation helped me better understand disparities in oral health based on socio‐economic status.	119 (72.6%)	124 (77.5%)
I am more likely to treat low‐income patients after this activity.	116 (70.7%)	105 (65.6%)
I am more likely to treat patients with Medicaid after this activity.	117 (71.8%)	97 (60.6%)

The persistence of changes in attitudes and evaluations are reported in Table 3. Mean poverty attitude scores increased by 9.4 points (SD = 16.6) between T_1_ and T_2_ but also declined by 2.3 points (SD = 6.0) between T_2_ and T_3_. Thus, H_1_ was supported: the improvement in poverty attitudes documented immediately after the simulation did change significantly 2 months (T_3_) later compared to T_2_. Although the T_3_ attitudes regressed toward the baseline mean, they remained significantly improved over baseline attitude scores. Regarding CAPS evaluations, the mean T_2_ evaluation score was 23.6 (SD = 5.5) of a possible 8 to 32 points. Unlike the changes in attitude scores, a decline in post‐simulation evaluations between T_2_ and T_3_ was not found, so H_2_ was not supported.

**TABLE 3:** Poverty attitudes and poverty simulation evaluation comparisons between baseline, post‐, and follow‐up surveys.

	T_1_ *M* (SD)	T_2_ *M* (SD)	T_3_ *M* (SD)	T_2_–T_1_ *M* (SD)	T_3_–T_1_ *M* (SD)	T_3_–T_2_ *M* (SD)
Poverty attitudes	64.1 (14.4) *n* = 169	73.5 (11.9) *n* = 162	71.7 (11.7) *n* = 159	−9.4 (16.6) *p* < 0.001, *n* = 162	−7.9 (18.3) *p* < 0.001, *n* = 159	−2.3 (6.0) *p* < 0.001 *n* = 153
Simulation evaluation		23.6 (5.5) *n* = 163	23.1 (5.4) *n* = 159			−0.5 (4.2) *p* = 0.146, *n* = 154

*Note*: Paired‐samples *t*‐tests and 2‐tailed tests of significance. Discrepancies in mean differences are attributable to missing (unpaired) data and to rounding.

Table 4 reports correlations between poverty attitudes and CAPS evaluations at different time points. Baseline (T_1_) poverty attitudes did not predict subsequent evaluations. However, T_2_ poverty attitudes correlated with T_2_ and T_3_ evaluations. Also, T_3_ poverty attitudes were correlated with T_2_ evaluations and T_3_ evaluations. These four correlations reflected medium effect sizes. Thus, H_3_ was partially supported: although baseline poverty attitudes did not predict CAPS evaluations, after the simulation attitudes and evaluations were positively correlated.

**TABLE 4:** Correlations between poverty attitudes and simulation evaluation.

	Simulation evaluation	
	T_2_ *r*	T_3_ *r*
T_1_ poverty attitude	−0.05 (*p* = 0.246, *n* = 163)	−0.04 (*p* = 0.307, *n* = 159)
T_2_ poverty attitude	0.33 (*p* < 0.001, *n* = 160)	0.45 (*p* < 0.001, *n* = 153)
T_3_ poverty attitude	0.35 (*p* < 0.001, *n* = 154)	0.44 (*p* < 0.001, *n* = 158)

*Note*: Pearson correlations and 1‐tailed tests of significance.

## 4 Discussion

This investigation surveyed students before a poverty simulation and then twice afterward to document changes in poverty attitudes and evaluations of the simulation. The results of this study are consistent with other studies, which have found that the majority of students show improved attitudes regarding poverty immediately after participating in a poverty simulation [17, 23, 24, 25]. In addition, this study aligned with other studies which found that 75% or more of the participants viewed the simulation as beneficial [17, 23, 24].

However, not all findings were positive. Our study aligned with a 2022 study which found that for some individuals the improved attitudes are not sustained over time [24]. Our study also aligned with a 2021 study which found that some students exhibited more negative attitudes about poverty after the simulation [25]. In our study, attitudes improved by a mean 7.9 points (SD = 18.3) between T_1_ and T_3_, which is impressive, but may obscure the finding that 34.6% of students reported a decrease in attitudes toward poverty during that time.

Any instructional initiative which results in poorer scores for more than a third of students should be reconsidered. Poverty simulations yield substantial benefits for the majority of participants, so it would help to know more about those who are not benefiting and what might be done either to enhance their simulation experience or to find an alternative instructional method that would facilitate better understanding of patients facing financial struggles. Regarding characteristics of those not benefitting, this study found baseline poverty attitudes did not predict evaluations, but respondents with less favorable attitudes toward poverty after the simulation were less likely to evaluate the simulation positively. Evaluation questions included self‐reports whether the simulation enhanced understanding of financial challenges, barriers to health, and oral health disparities related to socioeconomic status. A question also addresses students’ intention to treat economically disadvantaged patients in the future. So poorer post‐simulation poverty attitudes and CAPS evaluations suggest the potential for neutral or negative effects on patient oral health. Given these stakes, further research should be undertaken to determine whether any student characteristics can be identified before the simulation that would accurately predict negative attitudes after the simulation.

To extend the benefits of CAPS experienced by most students to the minority of students who are not meeting its learning objectives, different teaching strategies may be necessary. It would help to know why the simulation results in worse attitudes for this minority of students. This question has received little attention in existing studies. One possibility is that some dental students devalue behavioral sciences. A study of dental student attitudes toward communication skills found high variability in factors like the importance of mastering these skills and their relevance to success in dental school [37]. Another possibility is that students may appreciate behavioral sciences, yet prioritize clinical curricula. A Canadian study found dental students split on this topic, with the demands of other coursework leading some to reject the need for dedicated communication courses [38]. As one student in that study noted, “formal learning or training in communication will not make me respect people any more or any less” [38].

The timing may also have had a differential effect. Students in our simulation had an exam scheduled in another class in the afternoon, and it is conceivable that a minority resented a full morning of mandatory behavioral science instruction when their time might have been better spent. Political context also may have played a role, as these data were collected during a time of increasing polarization on diversity, equity, and inclusion initiatives at the federal, state, and professional levels, and this polarization may have affected some students’ experience of this course requirement [39]. Finally, the experience itself may have affected students differently. In their 2016 article, Browne and Roll documented the risks of teaching about poverty and of poverty simulations [26]—potentially perceived by some as acting poor for 3 hours and subsequently claiming to understand economic hardship.

If the types of students who have not historically benefitted can be identified and continue to participate in poverty simulations, additional or alternative strategies may improve their experience. Some strategic enhancements have been reported in the literature. Researchers at the University of Louisville elicited implementation suggestions from the low‐income volunteers who facilitated their poverty simulation [31]. Those suggestions included requiring more post‐simulation engagement with volunteers to hear their stories, requiring more time devoted to small‐group debriefings, and requiring experiences associated with poverty (e.g. walking to appointments, standing in social service lines, or staying in a shelter). Researchers at the University of Alabama at Birmingham recommended implementing several best practices in simulation, including training volunteers, pre‐briefing students in small groups and including psychological safety guidelines, and structured debriefings with trained volunteers [32]. Future research should experiment with these proposals and report the results.

For students who do not respond positively to CAPS, alternatives are also worth exploring. A 2017 study found CAPS and the online SPENT game both improved perceptions of poverty among pharmacy students [40]. A 2020 study found CAPS enhanced nursing students’ perceptions of poverty more than a Hunger Banquet experience did [41]. A 2021 study found a table‐top simulation had a more profound effect than CAPS on nursing students’ willingness to help and their empathy toward those in poverty [42]. A 2020 review compared elements of nine poverty simulations and advocated for the Experiential Poverty Exercise, which the authors developed [43]. The elements they recommended included interprofessional participation, immersive long‐term participation over weeks or months, alignment with poverty‐relevant course content, and debriefings and discussions [43]. Alternatives to poverty simulations might include service learning or panel presentations featuring patients in poverty. A 2022 cross‐sectional study found pre‐dental students who had experienced difficulty finding a dentist or getting an appointment expressed significantly higher intentions to serve underrepresented patient populations [44]. So, an experiential learning activity like trying to find a dentist who accepts Medicaid may make an impact. Research, however, has not yet identified an instructional method that reliably increases intentions to treat. Paradoxically, Valentine and Inglehart found that students who enjoyed treating Medicaid patients and were more confident in doing so actually were less likely to treat them in the future [45]. So, the above ideas and other innovations should be assessed for their impact on attitudes and intentions to treat patients in poverty.

This study provided a more detailed analysis of well‐documented poverty simulation attitude improvements by also measuring the scope of a problem which is rarely acknowledged: some students do not benefit or value these simulations, and their participation actually may be counterproductive. Our results indicate a vulnerability in predoctoral dental education, particularly related to CODA standard 2‐17, which all students are required to meet. If, as we found, poverty simulations result in a decline in attitudes for a subset of participants and, as we found, the positive impacts of poverty simulations abate over time, there are concerning implications for efforts to reduce healthcare disparities.

Strengths of this study include the use of anonymous responses to enhance validity and the addition of a follow‐up survey 2 months after the simulation to examine whether changed attitudes and evaluations persist over time. The results, however, may not be generalizable to other students at different points in their dental school careers or who have experienced different personal interactions with financial insecurity. Also, we did not investigate other influences which may have contributed to the decreased poverty attitude at T_3_ compared to T_2_. The results reflect the attitudes and evaluations of students in 2 years at one dental program, which limits generalizability. Further, the measures used were not psychometrically validated. Future research should integrate regular assessment using valid and reliable measures so that both positive and negative outcomes may be tracked. Enhanced assessment should also be used to compare different simulations and to compare poverty simulations to other forms of instruction. Future research should also seek to identify—before their participation—student characteristics indicating poverty simulations may be counterproductive. Finally, researchers should also assess other potential negative outcomes in addition to poorer attitudes and intentions to treat.

In conclusion, poverty simulations enhance poverty attitudes on average but potentially worsen attitudes among substantial minorities of students. Those with worse attitudes toward poverty are likely to value the simulation less and may require different teaching strategies.

## Conflicts of Interest

The authors declare no conflicts of interest.
